# Psychotic manifestations as the first presentation of Huntington’s disease: a case report

**DOI:** 10.1192/j.eurpsy.2026.12150

**Published:** 2026-07-31

**Authors:** M. S. Molina, E. Arroyo Sánchez, C. Díaz Mayoral, J. Gimillo Bonaque

**Affiliations:** 1 Psychiatry, Hospital Universitario Príncipe de Asturias; 2 Psychiatry, Hospital Blua Sanitas Valdebebas, Madrid, Spain

## Abstract

**Introduction:**

Huntington’s disease (HD) is a neurodegenerative disorder characterized by motor, cognitive, and psychiatric symptoms that are often misdiagnosed. We present a case of a patient with a purely psychotic debut and a family history of the disease.

**Objectives:**

To describe a clinical case of late-onset psychosis in the context of a family history of Huntington’s disease, to highlight the importance of considering this pathology in the differential diagnosis, even in the absence of obvious motor symptoms.

**Methods:**

A 44-year-old woman with a family history of Huntington’s Chorea (father and sister) was admitted due to a clinical picture of psychomotor agitation and persecutory delusional ideas. The psychopathological exploration revealed auditory hallucinations and structured delusional ideas. The CT scan showed frontal atrophy. Initial treatment consisted of olanzapine. For diagnostic confirmation, tests are recommended, such as the genetic test for the HTT gene mutation (CAG repeat expansion), a brain magnetic resonance imaging (MRI) to evaluate caudate and putamen nucleus atrophy (an early sign in Huntington’s disease), and a detailed neurological examination to detect subtle signs, such as alteration in oculomotor saccade, subtle dysarthria, or dystonia.

**Results:**

The clinical picture was compatible with a paranoid type psychotic disorder. The treatment with olanzapine resulted in a behavioral improvement, allowing for pharmacological de-escalation and the removal of physical restraint. The evolutionary report of the admission established the diagnostic suspicion of Huntington’s chorea as the basis of the clinical picture. The patient, a carrier of the HD gene, was discharged with follow-up in the parkinson’s and abnormal movement unit.

**Conclusions:**

This case report demonstrates the need for a comprehensive clinical history in patients with late-onset psychosis, as psychiatric manifestations can be the initial symptom of HD. It emphasizes the importance of close coordination between psychiatry and neurology for timely diagnosis and optimal therapeutic management. The approach should include symptomatic treatment, with a focus on avoiding typical antipsychotics like haloperidol and being cautious with some atypicals like Risperidone, as they can exacerbate motor symptoms. Pharmacological treatment should be guided by evidence; the drugs of choice are atypical antipsychotics such as Quetiapine or Clozapine, and in cases of affective symptoms, the inclusion of mood stabilizers like valproate, while lithium should be avoided to prevent the worsening of dyskinesia.

**Disclosure of Interest:**

None Declared

